# Effects of incubation temperature on the upper thermal tolerance of the imperiled longfin smelt (*Spirinchus thaleichthys*)

**DOI:** 10.1093/conphys/coae004

**Published:** 2024-02-10

**Authors:** Yuzo R Yanagitsuru, Florian Mauduit, Alexis J Lundquist, Levi S Lewis, James A Hobbs, Tien-Chieh Hung, Richard E Connon, Nann A Fangue

**Affiliations:** Department of Wildlife, Fish, and Conservation Biology, University of California, Davis, One Shields Avenue, Davis, CA, Yolo County, 95616, USA; Department of Wildlife, Fish, and Conservation Biology, University of California, Davis, One Shields Avenue, Davis, CA, Yolo County, 95616, USA; Department of Anatomy, Physiology, and Cell Biology, School of Veterinary Medicine, University of California, Davis, One Shields Avenue, Davis, CA, Yolo County, 95616, USA; Department of Wildlife, Fish, and Conservation Biology, University of California, Davis, One Shields Avenue, Davis, CA, Yolo County, 95616, USA; Department of Wildlife, Fish, and Conservation Biology, University of California, Davis, One Shields Avenue, Davis, CA, Yolo County, 95616, USA; Department of Wildlife, Fish, and Conservation Biology, University of California, Davis, One Shields Avenue, Davis, CA, Yolo County, 95616, USA; California Department of Fish and Wildlife, Bay-Delta IEP, 2109 Arch Airport Rd,Stockton, CA, San Joaquin County, 95206, USA; Fish Conservation and Culture Laboratory, Department of Biological and Agricultural Engineering, University of California, Davis, 17501 Byron Hwy, Byron, CA, Contra Costa County, 94514, USA; Department of Anatomy, Physiology, and Cell Biology, School of Veterinary Medicine, University of California, Davis, One Shields Avenue, Davis, CA, Yolo County, 95616, USA; Department of Wildlife, Fish, and Conservation Biology, University of California, Davis, One Shields Avenue, Davis, CA, Yolo County, 95616, USA

**Keywords:** Arrhenius breakpoint, cardiac performance, climate change, heart rate, larvae, San Francisco Estuary, thermal performance

## Abstract

Upper thermal limits in many fish species are limited, in part, by the heart’s ability to meet increased oxygen demand during high temperatures. Cardiac plasticity induced by developmental temperatures can therefore influence thermal tolerance. Here, we determined how incubation temperatures during the embryonic stage influence cardiac performance across temperatures during the sensitive larval stage of the imperiled longfin smelt. We transposed a cardiac assay for larger fish to newly hatched larvae that were incubated at 9°C, 12°C or 15°C. We measured heart rate over increases in temperature to identify the Arrhenius breakpoint temperature (T_AB_), a proxy for thermal optimum and two upper thermal limit metrics: temperature when heart rate is maximized (T_peak_) and when cardiac arrhythmia occurs (T_Arr_). Higher incubation temperatures increased T_AB_, T_peak_ and T_Arr_, but high individual variation in all three metrics resulted in great overlap of individuals at T_AB_, T_peak_ and T_Arr_ across temperatures. We found that the temperatures at which 10% of individuals reached T_peak_ or T_Arr_ and temperatures at which number of individuals at T_AB_ relative to T_peak_ (ΔN(T_AB,_T_peak_)) was maximal, correlated more closely with upper thermal limits and thermal optima inferred from previous studies, compared to the mean values of the three cardiac metrics of the present study. Higher incubation temperatures increased the 10% T_peak_ and T_Arr_ thresholds but maximum ΔN(T_AB,_T_peak_) largely remained the same, suggesting that incubation temperatures modulate upper thermal limits but not T_opt_ for a group of larvae. Overall, by measuring cardiac performance across temperatures, we defined upper thermal limits (10% thresholds; T_peak_, 14.4–17.5°C; T_Arr_, 16.9–20.2°C) and optima (ΔN(T_AB,_T_peak_), 12.4–14.4°C) that can guide conservation strategies for longfin smelt and demonstrated the potential of this cardiac assay for informing conservation plans for the early life stages of fish.

## Teaser

Using a common cardiac thermal performance assay, we identified temperature ranges that define the thermal optima and upper thermal limits of the larval life stage of the imperiled longfin smelt larvae. We demonstrated the utility of a cardiac assay for defining appropriate thermal conditions for a species of conservation concern.

## Introduction

Water temperature influences most biochemical and physiological processes of ectotherms, such as fishes, and is among the most important drivers of fish population distribution and survival (Brett, 1971; Reynolds and Casterlin, 1979). The individual physiological performances across temperatures and thermal tolerance limits of fishes have been linked to broader ecological processes, such as species distributions (Eliason *et al.,* 2011; Guo *et al.,* 2021) and population productivity (Moyano *et al.,* 2020), and have been applied to aquaculture programs to optimize culture methods (Brauner and Richards, 2020). As water temperatures continue to warm due to climate change, evaluating thermal tolerances and the influence of water temperature on fish physiological performance has become increasingly essential for the development of conservation strategies for imperiled fish species (Cooke *et al.,* 2021).

Temperature begins shaping the physiology of fishes as early as embryogenesis. A substantial body of research has demonstrated that, generally, higher incubation temperatures shift the thermal optimum for physiological performance and upper thermal tolerance limits higher at the juvenile and adult life stages (reviewed in Jonsson and Jonsson, 2014, 2019). For example, higher embryonic incubation temperatures improve swimming performance of adult zebrafish (*Danio rerio*) in warmer water through alterations in muscle fiber composition and the muscle transcriptome (Scott and Johnston, 2012). Similarly, higher incubation temperatures increased upper cardiac thermal tolerance of juvenile Atlantic salmon (*Salmo salar*) through alterations in myocardial morphology and the ventricular proteome (Muir *et al.,* 2022a, 2022b). There is comparatively less known about how incubation temperature affects the thermal physiology of the life stage immediately post-hatching, larvae. Some evidence suggests a similar pattern to juveniles and adults whereby higher incubation temperatures alter thermal tolerance by shifting their upper thermal limits higher (Dash *et al.,* 2021).

Fish larvae are considered to be among the most thermally sensitive life stages for fish (Killen *et al.,* 2007; Pörtner and Farrell, 2008; Pörtner and Peck, 2010). This life stage is characterized by extremely high mortality both in the wild and in captivity, which impacts the recruitment of a species in the wild (Hjort, 1914; Victor, 1986; Houde, 1994) and can impede the development of aquaculture programs (Holt *et al.,* 2007; DiMaggio *et al.,* 2013, 2017; Symonds *et al.,* 2014; Degidio *et al.,* 2017; Goetz *et al.,* 2021; Groover *et al.,* 2021). Therefore, targeted efforts to improve the survival of the larval stage could have a higher impact on a fish species' productivity. Temperature drives many of the factors that influence larval survival. For example, temperature affects growth rates, swimming ability, yolk utilization efficiencies, digestion and the energetic demands of development (Gunnarsson *et al.,* 2011; Mueller *et al.,* 2015; Yanagitsuru *et al.,* 2021; Lo *et al.,* 2022; McInturf *et al.,* 2022; Zillig et al., 2022). Indeed, temperature correlates strongly with the recruitment of some fish species (Ghil and Vautard, 1991; Galloway *et al.,* 1998; Sponaugle *et al.,* 2006) and explains a substantial amount of the observed variation in larval mortality rates for wild populations (Pepin, 1991). Evaluating the physiological performance of larval fish across temperatures, and how thermal history during the embryonic stage influences this, is thus a critical step towards developing plans to protect wild populations and improve methods for conservation aquaculture. For example, optimizing temperature for larviculture is essential for effective aquaculture programs (Shields, 2001), and the relationship between fish thermal tolerance and natural water temperature is a critical consideration for successful population supplementation efforts (Karppinen *et al.,* 2014; Yanagitsuru *et al.,* 2022).

Differential physiological performance across temperatures has been postulated to be driven by changes in the capacity to supply oxygen to meet the organism’s oxygen demand (Pörtner, 2001; Pörtner *et al.,* 2017). This capacity can be measured as aerobic scope, the difference between maximum and standard metabolic rates, and describes the scope for aerobically dependent activities such as feeding, growth and reproduction (Farrell *et al.,* 2008; Auer *et al.,* 2015a, 2015b). The temperature-dependent nature of aerobic scope can be described by the Fry aerobic scope curve (Fry and Hart, 1948), whereby aerobic scope is greatest at the organism's optimal temperature (T_opt_) and decreases at temperatures above or below T_opt_, until a critical temperature is reached where aerobic scope is zero (T_crit_), and only short-term survival is possible (Farrell, 2009). While aerobic scope is commonly measured for the juvenile and adult life stages of many fish species (Farrell, 2016), it may not be amenable to early life stages; comparatively fewer studies have measured aerobic scope in larvae due to their highly active states that prevent accurate measurements of standard metabolic rates and their poor response to the forced swimming protocols commonly used to measure maximum metabolic rates (Peck and Moyano, 2016).

While oxygen consumption from the external environment is used to calculate aerobic scope, oxygen delivery to tissues depends on cardiac output. As a result, aspects of cardiac output such as heart rate (*f*_H_) correlate well with aerobic scope across temperatures (Steinhausen *et al.,* 2008; Eliason *et al.,* 2011). Moreover, because *f*_H_ is a relatively straightforward aspect of cardiac output to measure compared to stroke volume, and is the primary determinant of delivering oxygen to tissue during acute temperature increases (Farrell, 1991, 2009; Gamperl *et al.,* 2011), measuring *f*_H_ in association with acute temperature increases provides a simple and rapid (a few days compared to a few weeks to measure aerobic scope) method for evaluating thermal performance in individual fishes (Casselman *et al.,* 2012), which is particularly important during the early life stages where ontogenetic changes in physiology can occur rapidly (Downie *et al.,* 2023). This cardiac assay relies on a well-characterized cardiac response to temperature to identify multiple indices of thermal performance. As temperature rises, maximum heart rate (*f*_Hmax_) increases exponentially until an Arrhenius breakpoint temperature (T_AB_) is reached, and as temperature increases beyond T_AB_, *f*_Hmax_ continues to rise until a temperature is reached that elicits maximum *f*_Hmax_ (T_peak_). Thereafter, further increases in temperature lead to cardiac arrhythmia, indicating the onset of cardiac failure, the temperature of which is termed T_Arr_. These three indices, T_AB_, T_peak_ and T_Arr_ provide the basis through which thermal performance is evaluated with this cardiac assay. T_AB_ has been correlated with T_opt_ for fishes when acclimation temperatures were relatively cool (Casselman *et al.,* 2012; Anttila *et al.,* 2013; Ferreira *et al.,* 2014). While the exact mechanism behind this correlation has not been empirically determined, T_AB_ represents the temperature where *f*_Hmax_, and therefore cardiac output, is maximized while heart rate increases proportionally with the increasing metabolic demands that acute temperature increases pose; this is to say, oxygen delivery to tissues is maximized at T_AB_. T_peak_ and T_Arr_, on the other hand, indicate the temperatures where cardiac output can no longer increase to match the higher metabolic demands incurred by higher temperature and the onset of cardiac failure, respectively. Given that T_crit_ is reliant on the failure of metabolic and cardiac performance, it is likely that T_peak_ and T_Arr_ provide the basis for T_crit_. Indeed, both metrics have been measured in other species to be just below T_crit_ and have therefore been used as measures of upper thermal tolerance (Casselman *et al.,* 2012; Anttila *et al.,* 2013). While more work is necessary to evaluate whether this cardiac assay method can be applied across species and acclimation temperatures, the rapid pace at which the assay can be conducted and its ability to measure multiple metrics of thermal performance within individual fish has garnered its attention, and it has become an increasingly common way to assess thermal performance in fishes (Casselman *et al.,* 2012; Chen *et al.,* 2013, 2015; Verhille *et al.,* 2013; Sidhu *et al.,* 2014; Drost *et al.,* 2016; Anttila *et al.,* 2017; Moyano *et al.,* 2020; Muir *et al.,* 2022a). Furthermore, this cardiac assay has a clear endpoint (i.e. cardiac arrhythmia) compared to the classical metric of T_crit_, critical thermal maximum, for which the loss of equilibrium endpoint can be difficult to ascertain in slow-moving larvae that often have undeveloped swim bladders and sometimes lay at the bottom of containers (Moyano *et al.,* 2017). In summary, by measuring *f*_Hmax_ over acute temperature increases, T_AB_ can be used as a proxy for T_opt_, and T_crit_ can be approximated, albeit with an underestimation, with T_peak_ and T_Arr_.

Longfin smelt (*Spirinchus thaleichthys*) is an imperiled forage fish species that is distributed along the Pacific coast of North America from California to Alaska. While some landlocked populations exist in Washington, United States and British Columbia, Canada, most populations are anadromous with adults migrating from coastal marine habitats between October and April to streams and estuaries to spawn (Moyle, 2002; Garwood, 2017; Lewis *et al.,* 2020). The timing of outmigration for longfin smelt is unclear but is thought to occur in the summer months (beginning June) as water temperatures rise (Rosenfield and Baxter, 2007). The San Francisco Estuary (SFE) population of longfin smelt is at the southernmost range of the species and is likely a genetic source population for several Northern coastal populations of longfin smelt (Garwood, 2017; Saglam *et al.,* 2021). They were once one of the most abundant fish species in the SFE but are now reduced to approximately 1% of their historic (pre-1980s) abundance, have been officially proposed for listing as a federally endangered species, and are at risk of extirpation (Sommer *et al.,* 2007; Hobbs *et al.,* 2017; USFWS, 2023). Despite more than 50 years of population monitoring surveys, there is limited knowledge about the biology and physiology of the species and, in turn, a lack of understanding regarding how the species responds to variations in environmental conditions such as temperature (Hobbs *et al.,* 2017). As temperatures continue to rise in the SFE due to climate change, and the particularly large spatial extent of temperature increases across the SFE during the longfin smelt spawning season, it is imperative to evaluate the upper thermal tolerance of the early life stages of longfin smelt and their capacity to acclimate to higher temperatures (Bashevkin *et al.,* 2022).

To begin evaluating the upper thermal tolerance of longfin smelt larvae and how developmental temperatures could influence it, we took advantage of a well-documented cardiac response to temperature to measure T_AB_ as a proxy for T_opt_, and T_peak_ and T_Arr_ as measures of upper thermal limits. Nearly all previous studies using the cardiac assay described above have been conducted on post-larval life stages of fishes, so we therefore transposed the methods developed for larger fish to newly hatched larvae. We expected T_AB_, T_peak_ and T_Arr_ to increase with incubation temperature. We also predicted that longfin smelt yolk-sac larvae would have a T_AB_ between 8°C and 12°C as larval longfin smelt are most abundant at these temperatures in the SFE (Grimaldo *et al.,* 2017) and because a recent laboratory study has shown improved growth performance at 9°C and 12°C (Yanagitsuru *et al.,* 2021). Finally, we predicted that T_peak_ and T_Arr_ would be around 15°C to 16°C as larval longfin smelt abundance declines in the SFE after 16°C and because larvae exhibited reduced growth performance at 15°C (Grimaldo *et al.,* 2017; Yanagitsuru *et al.,* 2021).

## Materials and Methods

### Broodstock and embryo collection

Longfin smelt broodstock were collected between November and December 2019 by the US Fish and Wildlife Services Chipps Island trawl with a midwater trawl, the UC Davis Fish Conservation and Culture Laboratory (FCCL) using a lampara net in the Sacramento San Joaquin Delta, and the UC Davis Otolith Geochemistry and Fish Ecology Laboratory using an otter trawl in the Alviso Marsh in South San Francisco Bay. Broodstock collections were approved under California Department of Fish and Wildlife MOU ID: Hobbs_LFS_2021 and Specific Use Permit IDs: S-191990002-19, 199-001, and D-0021521915-6. Collected fish were transported to the FCCL where they were held at 12°C in 10 parts per-thousand (ppt) water and fed live adult artemia. As soon as a female became gravid, a single female and male pair were strip-spawned into a plastic bowl where eggs and milt were mixed in freshwater from the Delta (0.2 ppt) at 12°C. A subset of 300 fertilized embryos from each clutch to be used for experiments were then transported to the UC Davis Fish Conservation Physiology Laboratory in conical tubes within coolers, lightly chilled with ice packs (approximately 1.5 hours transport time). Water temperatures within conical tubes were between 10.9°C and 12.1°C upon arrival. Subsets of 300 embryos from five single pair crosses, for a total of 1500 embryos, were incubated for experiments between December 2019 and January 2020.

### Incubation temperatures

Embryos from each cross were distributed amongst three experimental incubation temperatures that spanned the range of temperatures that the early life stages of longfin smelt may experience in the SFE: 9°C, 12°C or 15°C. Embryos from each clutch were divided into three groups of 100 (one for each incubation temperature) within 270 ml plastic bowls that were filled with freshwater sourced from a well (0.4 ppt, pH 8.4–8.6) and floated in water baths at 12°C. Temperatures in each water bath were adjusted at a rate of approximately 0.5°C∙h^−1^ until experimental temperatures were reached. Water temperatures within bowls (9°C: 8.8 ± 0.3°C; 12°C: 12.0 ± 0.3°C; 15°C: 14.8 ± 0.4°C; mean ± SD) were monitored daily with an Omega HH82A digital thermocouple thermometer (Omega Engineering Inc., Norwalk, CT, USA), and water quality in each bowl was maintained with ~ 75% water changes daily. To prevent the spread of diseases, embryos afflicted by fungus, and dead embryos indicated by a milky yellow coloration similar to that observed in dead delta smelt embryos (Tsai *et al.,* 2021), were removed daily. We did not record hatching rates but expect that there was slightly lower hatching in the 15°C incubation group (Yanagitsuru *et al.,* 2021). Once hatched, individual larvae were gently pipetted into plastic bowls floating in water baths at their incubation temperature. Because more individuals hatched than could fit under the experimental setup (see Section 2.3), a subset of hatched individuals were randomly selected to be experimented upon the following day (one day post hatch).

### Heart rate measurements

We developed an experimental setup that allowed us to transpose the cardiac assay initially developed by Casselman *et al.* (2012) for larger fish, to larvae. The cardiac assay experimental setup consisted of a petri dish fitted with a glass plate for placing larval fishes. Petri dishes were additionally perforated on the bottom and fitted with a 335-μm nylon mesh within a water bath. The perforations and nylon mesh allowed gentle water exchange from the water bath to hasten temperature equalization within the dish and water bath without disturbing the larvae. Water baths were temperature-controlled via recirculation with a Delta Star air-cooled heat pump (Aqualogic Inc., San Diego, CA, USA). The experimental setup was placed under a Leica S8APO stereomicroscope (Leica Microsystems, Chicago, IL, USA) mounted with a Canon EOS Rebel T6 SLR camera (Canon, Tokyo, Japan) to record videos of transparent larvae, which had hearts visible (Supplementary Fig. 1; Supplementary Video 1).

Prior to the start of an experiment, water bath temperature was set to 9°C. To ensure that larvae would not move during recordings, MS-222 was mixed into bath water to a concentration of 0.08 g∙L^−1^, which preliminary trials found to be the lowest concentration necessary to prevent spontaneous body movements for the duration of experiments (~5 hours). This MS-222 concentration is well below that which has been tested and found to have no effect on heart rate in the larvae of several other fish species (Moyano *et al.,* 2020). Decrease in well water pH after addition of MS-222 at this dose was minimal (≤0.01 pH unit) likely due to the hardness of well water so pH adjustment with addition of NaHCO_3_ was not necessary (Allen and Harman, 1970). Once larvae were anaesthetized, up to 30 larvae were positioned within the petri dish under the microscope and left to acclimate for 1 hour. Water bath temperatures were then increased in a stepwise fashion from 9°C to 25°C at 2-°C increments between 9°C and 19°C (9, 11, 13, 15, 17, 19°C) and 1°C increments between 19°C and 25°C (19°C, 20°C, 21°C, 22°C, 23°C, 24°C, 25°C) at a rate of approximately 0.6°C∙min^−1^ between temperature steps. At each temperature step, larvae were exposed to the new temperature for 5 min and then filmed at 30 frames s^−1^ for 2.5 minutes under a bright light before increasing the temperature again (i.e. larvae were exposed to the new temperature step for a total of 7.5 min). Following the final recording at 25°C, larvae were euthanized with MS-222 overdose (0.5 g∙L^−1^ buffered to pH 8.0). A total of 121, 152 and 134 individuals were video recorded at each of the twelve temperature steps for the 9°C, 12°C and 15°C groups, respectively, for a total of 407 larvae. Therefore, a total of 4884 (i.e. 407 fish * 12 temperatures) videos of larval hearts were recorded. Heart rates were not measured in videos where hearts had reached arrhythmia.

DanioScope (Noldus, Wageningen, Netherlands), an activity analysis software that measures the frequency of activity spikes (i.e. inter-beat intervals) to calculate *f*_H_ within selected areas, was used to measure *f*_H_ for most individuals (Supplementary Fig. 2). The first 30 s of video were excluded from analyses due to camera instability and *f*_H_ was measured in the 2 min between 0:30 and 2:30 at each temperature. Poor focus in some videos on the hearts of some larvae prevented DanioScope from recognizing activity spikes. For the larvae in these videos, *f*_H_ was measured with human visual observation (method described below). Of the 4884 larval heart videos recorded (one video per temperature per larva), 238 hearts (4.9% of heart rates) were measured with human visual observation. Because this software has not previously been used with longfin smelt, we first validated this software for the species by comparing the *f*_H_ measured with Danioscope and with human visual observation with 10 larvae. To measure *f*_H_ visually, the video segment from 0:30 to 2:30 for the 10 larvae was split into seven 15-s clips using Bandicut video editing software (Bandicam Company, Irvine, CA, USA). A single observer counted the number of heart beats in each clip and divided it by 0.25 min to calculate beats per minute. *f*_H_ for an individual was determined as the average of the *f*_H_ calculated in the seven clips. Automatic recordings of *f*_H_ with DanioScope strongly correlated with those obtained visually (*t*_8_ = 98.0, *P* < 0.001, R^2^ = 0.999), validating the accuracy of this software for longfin smelt larvae (Supplementary Fig. 3).

**Figure 1:**
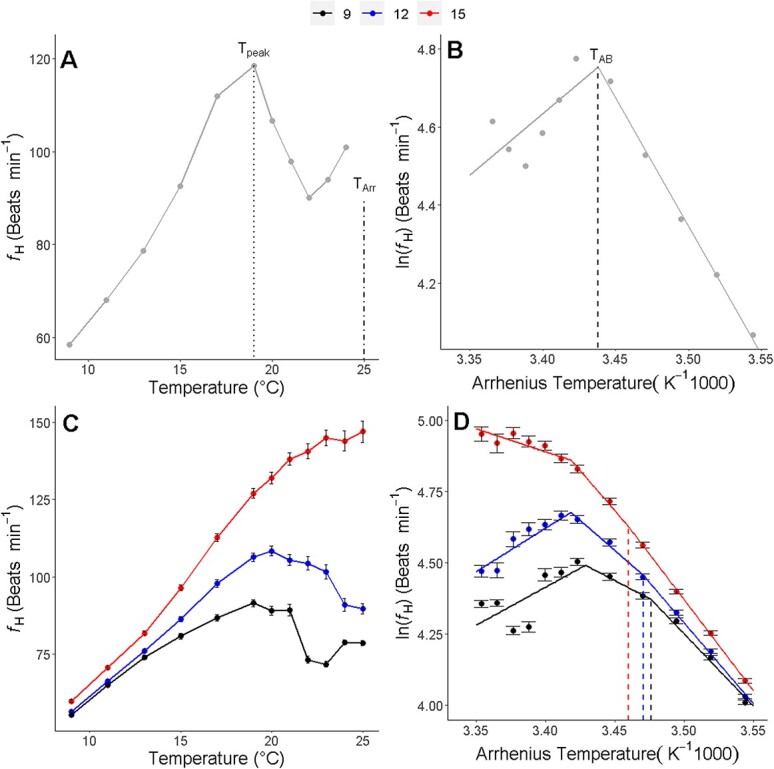
The effect of increasing temperature on heart rate (*f*_H_) of longfin smelt larvae. Representative plot of heart rate with temperature (A) and Arrhenius plot of *f*_H_ with increasing temperatures (B) for an individual larva. Average heart rate with temperature for all individuals (C) and Arrhenius plots of *f*_H_ with increasing temperatures (D) for different incubation temperature groups. Regression lines for representative Arrhenius plots are divided into two segments, representing the method by which Arrhenius breakpoint temperatures (T_AB_) were determined for each individual. Unlike individual Arrhenius plots, grouped Arrhenius plots were best fit as 3 segments due to variation in post-T_AB_ changes in *f*_H_ across individuals. Dotted line indicates temperature where heart rate peaks (T_peak_), dash-dotted line indicates temperature where cardiac arrhythmia was first observed (T_Arr_), and dashed line indicates the T_AB_. Data presented as mean ± SEM.

Using the *f*_H_ measured for each individual across stepwise increases in temperature, we measured T_AB_, T_peak_ and T_Arr_. T_AB_ was determined in R 3.6.3 (R Core Team, 2013) by plotting the log_10_ of *f*_H_ over the inverse of temperature (1000 K^−1^) (Fig. 1) and using the ‘segmented’ package (Muggeo, 2017), which uses piece-wise linear regression to determine breakpoints where linear relationships change. T_peak_ was determined by identifying the temperature at which the highest *f*_H_ was measured for an individual. T_Arr_ was determined as the temperature at which cardiac arrhythmia was first observed, which could be identified from Danioscope activity plots (Supplementary Fig. 2B) when activity spikes were discontinuous (Supplementary Fig. 2C; Supplementary Video 2). When activity spikes were discontinuous, videos were verified visually for arrhythmia. We also measured the initial *f*_H_ (*f*_H0_), *f*_H_ at T_peak_ (peak heart rate, *f*_Hpeak_), and the rate of increase in *f*_H_ with temperature prior to T_AB_ (d*f*_H_/dT) by calculating the slope of the linear relationship between *f*_H_ and temperature prior to T_AB_, for each individual. Finally, we calculated the difference between *f*_Hpeak_ and *f*_H0_ (∆*f*_Hpeak-H0_) as an indicator of cardiac scope.

Almost all previous studies on fish using this cardiac assay have been conducted on later life stages where it is recommended to pharmacologically induce *f*_Hmax_ with intraperitoneal injections of atropine sulphate and isoproterenol (e.g. Casselman *et al.,* 2012). For very small larval fish (<7 mm length, < 50 μg mass; Yanagitsuru *et al.,* 2021), it is exceedingly difficult to perform such injections and cardioactive drugs were thus not used in this study (Drost *et al.,* 2016). However, pharmacological agents have relatively minor effects on the cardiac metrics measured in fish (Casselman *et al.,* 2012) and atropine sulphate dissolved in water had either no effect or decreased *f*_H_ depending on concentration in larval Arctic cod (*Boreogadus saida*) (Drost *et al.,* 2016). Furthermore, bright lights, as used during our experiments, are known to induce hyperactivity in the larval stage of a similar fish species; delta smelt (Mundy *et al.,* 2020). We thus report *f*_H_ with the expectation that *f*_Hmax_ had been reached.

### Statistical analyses

All statistical analyses were performed using R 3.6.3 (R Core Team, 2013). The validation of DanioScope measurements was analysed using a linear regression with *f*_H_ measured with DanioScope as the response variable and *f*_H_ measured visually as the fixed effect. *f*_H0_, *f*_Hpeak_, ∆*f*_Hpeak-H0_, d*f*_H_/dT, T_AB_, T_peak_ and T_Arr_ were analysed separately as response variables using linear mixed effects models with incubation temperature as a fixed effect and clutch ID as a random effect. The significance of random effect was determined with a likelihood ratio test. Tukey’s multiple comparison post-hoc test was used to analyse differences between each pair of incubation temperature within clutches using the ‘lsmeans’ package (Lenth, 2016). Effect sizes as measured by marginal and conditional R^2^ values were calculated with the ‘r.squaredGLMM’ function from the ‘MuMIn’ package (Nakagawa and Schielzeth, 2013; Johnson, 2014). We generated a kernel density plot for each incubation temperature group to visualize the proportion of individuals at T_AB_ across temperatures (Fig. 4) using ‘geom_density’ (Wickham *et al.,* 2016). We then extracted the values from this density plot to estimate the proportion of individuals at T_AB_ across temperatures. We additionally fit a logistic model on the proportion of individuals that reached T_peak_ and T_Arr_ with incubation temperature, stepwise temperature, and their interactions as fixed effects and clutch ID as a random effect. We estimated the temperatures at which 10%, 50% and 95% of individuals would have their heart rates peak or become arrhythmic with the ‘dose.p’ function in R (Venables and Ripley, 2002). Finally, we fit the difference between the number of individuals at T_AB_ and T_peak_ (∆N(T_AB,_T_peak_), and T_AB_ and T_Arr_ (∆N(T_AB,_T_Arr_) to generalized additive models (GAMs) with temperature as a predictor variable using the ‘mgcv’ package (Wood, 2017). The GAMs were fit with a Gaussian distribution and thin plate regression spline smoothing functions. The k value was set to 4 and 9, for T_peak_ and T_Arr_, respectively. Statistical significance was accepted at *P* < 0.05. All values are reported as mean ± SEM unless otherwise specified.

## Results

### Heart rate

Incubation temperature had significant effects on *f*_H0_, *f*_Hpeak_, ∆*f*_Hpeak-H0_ and d*f*_H_/dT (Table 1). Averaged across clutches, *f*_H0_, *f*_Hpeak_ and ∆*f*_Hpeak-H0_ increased with incubation temperatures but d*f*_H_/dT was only higher in the 15°C group, and there was no difference between the 9°C and 12°C groups. The random effect clutch ID had high variance (Supplementary Table 1), and a large difference between conditional and marginal R^2^ values for *f*_H0_, *f*_Hpeak_ and scope (Table 1). This was reflected in the relationships between *f*_H0_, *f*_Hpeak_ and ∆*f*_Hpeak-H0_, which were more variable across clutches for each incubation temperature (Table 2).

**Table 1:** Parameter estimates for fixed effects, marginal (R^2^m) and conditional R^2^ (R^2^c) values of linear mixed models for cardiac function metrics. See Supplementary Table 1 for parameter estimates of random effects. Bolded *P* values indicate statistical significance (*P* < 0.05).

Metric	Parameter	Estimate	SE	Df	*t* value	*P* value	R^2^m	R^2^c
Initial heart rate (*f*_H0_)	Intercept	48.87	1.66	13.66	29.51	**<0.001**		
	Incubation	0.64	0.09	402.77	7.39	**<0.001**	0.085	0.383
Peak heart rate (*f*_Hpeak_)	Intercept	5.12	7.56	12.70	0.68	0.510		
	Incubation	8.68	0.39	400.72	22.53	**<0.001**	0.459	0.647
Difference between initial and peak heart rate (∆*f*_Hpeak-H0_)	Intercept	−43.63	6.39	16.53	−6.83	**<0.001**		
	Incubation	8.03	0.36	400.98	22.44	**<0.001**	0.482	0.626
Rate of increase in heart rate prior to Arrhenius breakpoint temperature (d*f*_H_/dT)	Intercept	−4.77	0.40	261.47	−11.94	**<0.001**		
	Incubation	−0.11	0.03	402.92	−3.62	**<0.001**	0.032	0.050
Arrhenius breakpoint temperature (T_AB_)	Intercept	9.13	0.73	70.84	12.56	**<0.001**		
	Incubation	0.63	0.05	399.94	11.98	**<0.001**	0.252	0.317
Temperature where heart rate reaches a maximum (T_peak_)	Intercept	12.20	0.56	127.45	21.95	**<0.001**		
	Incubation	0.57	0.04	404.08	13.64	**<0.001**	0.310	0.346
Temperature where arrhythmia first occurs (T_Arr_)	Intercept	15.16	0.50	55.24	30.60	**<0.001**		
	Incubation	0.51	0.04	353.45	14.48	**<0.001**	0.355	0.427

**Table 2:** Metrics of cardiac function: initial heart rate (*f*_H0_), peak heart rate (*f*_Hpeak_), difference between initial and peak heart rate (∆*f*_Hpeak-H0_), rate of increase in heart rate with temperature prior to Arrhenius breakpoint temperature (d*f*_H_/dT), Arrhenius breakpoint temperature (T_AB_), temperature where heart rate reaches a maximum (T_peak_), and temperature where arrhythmia first occurs (T_Arr_) at different incubation temperatures for each clutch. *P* values for pairwise comparisons between incubation temperatures are listed for each cardiac function metric. Bolded *P* values indicate statistically significant (*P* < 0.05) differences among clutches and incubation temperature groups.

	Temp (°C)	Clutch 1	Clutch 2	Clutch 3	Clutch 4	Clutch 5	Average
*f*_H0_ (beats min^−1^)	9	57.4 ± 0.4	58.2 ± 1.3	52.9 ± 0.4	48.9 ± 0.7	55.9 ± 0.6	55.5 ± 0.4
	12	58.3 ± 0.4	61.2 ± 0.6	55.9 ± 0.6	54.6 ± 1.4	52.3 ± 1.0	56.9 ± 0.4
	15	60.0 ± 0.7	63.1 ± 1.2	59.8 ± 0.4	52.5 ± 0.6	54.2 ± 1.4	59.8 ± 0.5
*f*_Hpeak_ (beats min^−1^)	9	102.4 ± 2.0	97.0 ± 3.0	75.6 ± 1.1	68.8 ± 2.3	85.6 ± 1.4	87.9 ± 1.4
	12	115.9 ± 2.1	127.1 ± 3.1	98.5 ± 3.1	96.8 ± 6.0	98.4 ± 3.4	109.2 ± 1.6
	15	132.5 ± 3.7	159.5 ± 6.0	138.7 ± 2.5	101.7 ± 2.2	128.6 ± 11.5	140.9 ± 2.5
∆*f*_Hpeak-H0_ (beats min^−1^)	9	44.9 ± 1.8	38.9 ± 2.7	22.7 ± 0.8	19.2 ± 2.2	29.7 ± 1.3	32.4 ± 1.1
	12	57.6 ± 2.0	65.9 ± 2.9	42.6 ± 2.8	42.3 ± 6.0	46.0 ± 3.4	52.2 ± 1.5
	15	72.5 ± 4.0	96.3 ± 5.3	79.1 ± 2.5	49.2 ± 2.0	74.3 ± 11.5	81.1 ± 2.2
d*f*_H_/dT (beats min^−1^∙°C^−1^)	9	5.8 ± 0.2	6.7 ± 0.5	5.4 ± 0.1	4.8 ± 0.2	6.1 ± 0.2	5.9 ± 0.1
	12	6.3 ± 0.1	6.2 ± 0.1	6.3 ± 0.3	5.7 ± 0.8	5.7 ± 0.4	6.0 ± 0.1
	15	5.9 ± 0.3	6.8 ± 0.3	6.6 ± 0.3	6.5 ± 0.1	7.3 ± 0.9	6.6 ± 0.1
T_AB_ (°C)	9	16.5 ± 0.3	14.7 ± 0.3	13.8 ± 0.3	14.1 ± 0.9	14.2 ± 0.5	14.7 ± 0.2
	12	17.7 ± 0.4	17.9 ± 0.3	15.3 ± 0.4	16.8 ± 0.6	16.9 ± 0.5	17.0 ± 0.2
	15	18.8 ± 0.5	19.4 ± 0.3	17.9 ± 0.5	16.5 ± 0.7	18.0 ± 0.8	18.4 ± 0.2
T_peak_ (°C)	9	18.7 ± 0.3	17.6 ± 0.3	16.7 ± 0.2	16.6 ± 0.7	16.7 ± 0.2	17.3 ± 0.2
	12	19.1 ± 0.4	20.4 ± 0.3	18.3 ± 0.3	18.9 ± 0.5	19.9 ± 0.5	19.5 ± 0.2
	15	20.3 ± 0.4	21.4 ± 0.3	20.6 ± 0.3	19.4 ± 0.4	20.2 ± 0.8	20.7 ± 0.2
T_Arr_ (°C)	9	21.0 ± 0.2	20.1 ± 0.2	19.4 ± 0.2	18.6 ± 0.5	19.5 ± 0.2	19.8 ± 0.1
	12	22.1 ± 0.3	22.3 ± 0.2	20.4 ± 0.2	20.8 ± 0.4	21.3 ± 0.3	21.4 ± 0.1
	15	22.4 ± 0.4	23.3 ± 0.2	22.9 ± 0.3	21.8 ± 0.4	22.3 ± 0.5	22.8 ± 0.2

### Cardiac indices of thermal performance

**Figure 2:**
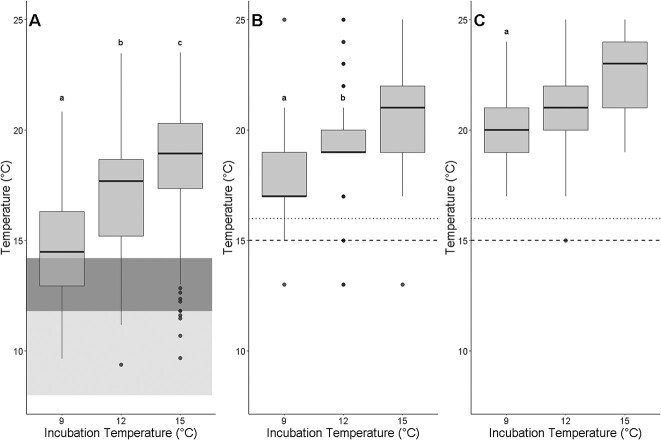
Median cardiac metrics do not correlate with inferred optimal temperatures or upper thermal limits. Arrhenius breakpoint temperature (T_AB_) (A), temperature where heart rate reaches a maximum (T_peak_) (B), and temperature where arrhythmia first occurs (T_Arr_) (C) of one day post-hatch longfin smelt larvae incubated at and held for one day at different temperatures. Boxplots represent median, first and third quartiles of cardiac performance metrics. Shaded dark gray area indicates the temperature range in which longfin smelt are cultured at the Fish Conservation and Culture Laboratory (Supplementary Table 3) and light gray shaded area indicates the temperatures where longfin smelt larvae are most abundant in the San Francisco Estuary. Dotted lines indicate the temperature beyond which longfin smelt larvae are no longer observed in the field and dashed lines indicate the temperature where reduced larval growth rates have been measured. Different letters indicate statistical significance between incubation temperatures within each cardiac performance metric.

Incubation temperature had a significant effect on T_AB_, T_peak_ and T_Arr_ (Table 1). All three metrics increased with incubation temperatures (Fig. 2). The random effect clutch ID had low variances and small differences between conditional and marginal R^2^ values for all three metrics (Table 2). This was reflected in the small interclutch variation in the response to incubation temperature for all three metrics but the same trend whereby higher incubation temperatures resulted in higher T_AB_, T_peak_ and T_Arr_ across clutches (Table 2). It should be noted that there were three individuals where T_AB_ could not be calculated because a breakpoint was not present prior to the individual becoming arrhythmic; these individuals were not included in the T_AB_ dataset. Additionally, not all individuals experienced arrhythmia within the range of temperatures tested (described below) and thus our measure of T_Arr_ is likely to be slightly underestimated.

**Figure 3:**
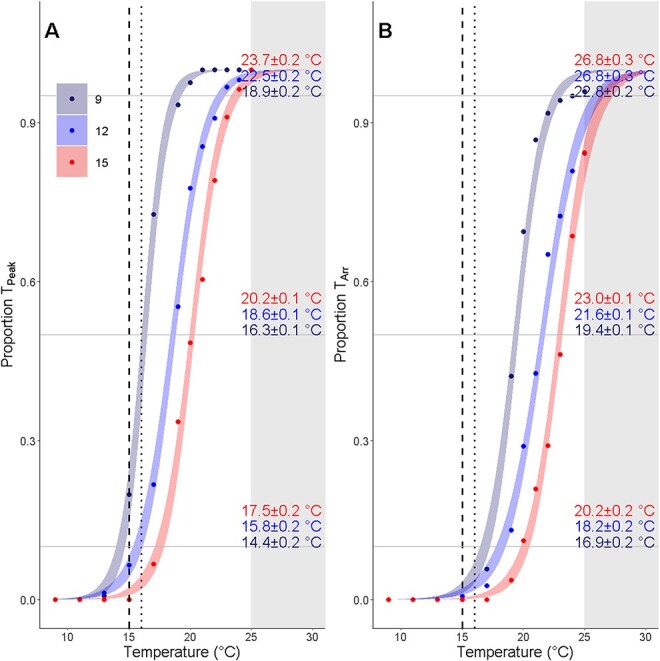
The 10% threshold for T_peak_ and T_Arr_ correlate more closely with indicators of upper thermal limits measured by previous studies. Proportion of individuals reaching temperatures where heart rate reaches a maximum (T_peak_) (A) and where arrhythmia first occurs (T_Arr_) (B) as test temperatures increased. Embryos were incubated at 9°C, 12°C, and 15°C. Gray shaded areas indicate temperatures beyond those tested experimentally and are thus predictions of the models. Horizontal lines indicate 10, 50 and 95% thresholds and temperatures listed correspond to the temperature at which these thresholds were met for each incubation temperature. Dotted lines indicate the temperature beyond which longfin smelt larvae are no longer observed in the field and dashed lines indicate the temperature where reduced larval growth rates have been measured.

There was a significant effect of temperature and incubation temperature, but not their interaction on the proportion of individuals that reached T_peak_ or T_Arr_ (Table 3). Notably, there was extremely high individual variation in cardiac indices regardless of incubation temperature. For 9°C, 12°C and 15°C incubation temperatures, T_AB_ ranged between 9.7°C to 20.8°C, 9.4°C to 23.5°C and 9.7°C to 23.5°C, and T_Arr_ ranged between 17°C to 24°C, 15°C to 25°C and 19–25°C for 9°C, 12°C and 15°C incubation groups, respectively. T_peak_ ranged between 13°C to 25°C for all incubation temperatures. Logistic curves showed that the temperature at which 10, 50 and 95% of the proportion of individuals that had reached T_peak_ or T_Arr_ increased with incubation temperature (Fig. 3). This was reflected in the significantly higher percentage of arrhythmic individuals by 25°C for the 9°C incubation temperature compared to the 12°C and 15°C incubation temperatures (F_2,413_ = 4.93, *P* = 0.009; 9–12°C: *P* = 0.007, 9–15°C: *P* = 0.036, 12–15°C: *P* = 0.954) where the proportion of arrhythmic individuals by 25°C was 95.8 ± 1.8, 82.1 ± 3.0 and 84.3 ± 3.2% for 9°C, 12°C and 15°C incubation temperatures, respectively.

**Table 3:** Parameter estimates, odds ratios, marginal (R^2^m) and conditional R^2^ (R^2^c) values of generalized mixed models for the proportion of individuals at T_peak_ and T_Arr_ across temperatures for different incubation temperatures as depicted in Fig. 3. Odds ratios were calculated as the exponential of parameter estimates. R^2^ values were calculated using the delta method. See Supplementary Table 2 for parameter estimates of random effects. Bolded *P* values indicates statistical significance (*P* < 0.05).

Metric	Parameter	Estimate±SE	z-value	*P* value	Odds Ratio	R^2^m	R^2^c
Temperature where heart rate reaches a maximum (T_peak_)	Intercept	−10.17 ± 2.32	−4.39	**<0.001**			
	Temperature	0.92 ± 0.12	7.40	**<0.001**	2.52	0.836	0.843
	Incubation	−0.50 ± 0.19	−2.59	**0.010**	0.61		
	Temperature x Incubation	−0.003 ± 0.01	−0.35	0.725	1.00		
Temperature where arrhythmia first occurs (T_Arr_)	Intercept	−10.31 ± 2.50	−4.12	**<0.001**			
	Temperature	0.75 ± 0.12	6.40	**<0.001**	2.12	0.783	0.793
	Incubation	−0.52 ± 0.21	−2.47	**0.014**	0.59		
	Temperature x Incubation	0.002 ± 0.01	0.23	0.821	1.00		

**Figure 4:**
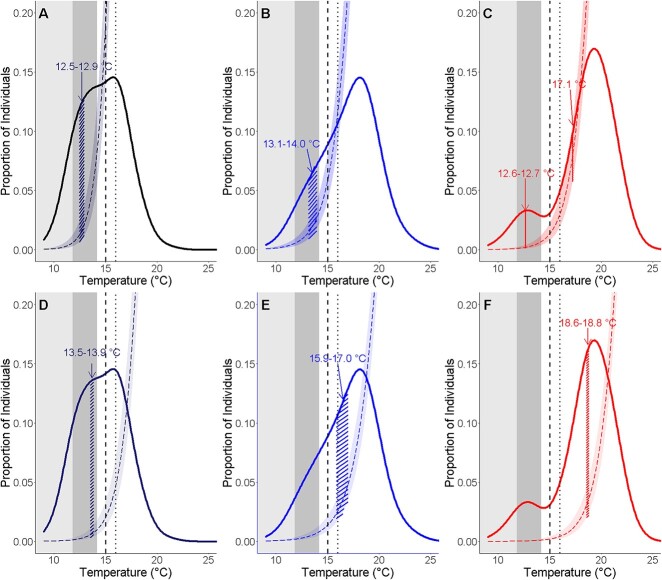
Overlays of proportion of individuals at Arrhenius breakpoint temperatures (T_AB_) and temperatures where heart rate is maximized (T_peak_) and experience arrhythmia (T_Arr_) reveal a temperature range where the proportion of individuals at T_AB_ relative to T_peak_ or T_Arr_ is maximized. Overlay of the proportion of individuals at T_AB_ and that have reached T_peak_ (A-C) and T_Arr_ (D-F) for 9°C (A,D), 12°C (B,E), and 15°C (C,F) incubation temperature groups. Smooths of proportion of individuals at T_AB_ are kernel density estimates of proportion of individuals at T_AB_. Dashed and dotted curves are logistic curves of the proportion of individuals at T_peak_ and T_Arr_. Logistic curves are the same as in Fig. 3. Hashed areas under curve indicate the temperature ranges where T_AB_-T_peak_ and T_AB_-T_Arr_ are maximized. Dark gray shaded area indicates temperature range that longfin smelt larvae are cultured at the Fish Conservation and Culture Laboratory (Supplementary Table 3), and light gray shaded areas indicate temperatures where larvae are most abundant in the San Francisco Estuary. Dotted lines indicate the temperature beyond which longfin smelt larvae are no longer observed in the field and dashed lines indicate the temperature where reduced larval growth rates have been measured.

The proportions of individuals that were at T_AB_ across temperatures were distributed in a bell-shaped curve for 9°C and 12°C, and a bimodal distribution for 15°C (Fig. 4). The temperatures where the estimated proportion of individuals at T_AB_ were within 90% of the maximal proportion of individuals at T_AB_ ranged between 13.1°C to 16.6°C, 17.0°Cto 19.0°C, and 18.4°C to 20.3°C, for 9°C, 12°C and 15°C incubation temperatures, respectively. The T_AB_ curve for the 15°C incubation temperature also had a second smaller peak at 12.8°C. Because there were some individuals reaching T_peak_ or T_Arr_ simultaneous to other individuals at T_AB_, overlaying logistic curves for T_peak_ and T_Arr_ with the T_AB_ curves revealed a range of temperatures where there are more individuals at T_AB_ relative to T_peak_ or T_Arr_ (Fig. 4). Calculating ∆N(T_AB_,T_peak_) found that the temperatures where the proportion of individuals at T_AB_ was maximized relative to T_peak_ ranged between 12.5°C to 12.9°C and 13.4°C to 14.4°C for 9°C and 12°C incubation temperatures, respectively (Fig. 4A). For the 15°C incubation temperature, there were two ranges where this difference was maximized: 12.6°C to 12.7°C and 17.2°C. Maximum values of ∆N(T_AB_,T_peak_) trended towards lower values with higher incubation temperatures; maximum ∆N(T_AB_,T_peak_) for 9°C, 12°C and 15°C were 0.11, 0.05 and 0.03, respectively (Fig. 4A–C). ∆N(T_AB-_T_Arr_) was maximized between 13.6°C to 13.9°C, 16.2°C to 16.9°C and 18.6°C to 18.8°C, for 9°C, 12°C and 15°C incubation temperatures, respectively (Fig. 4D–F). Similarly, maximum ∆N(T_AB_,T_Arr_) trended towards lower values with higher incubation temperatures; maximum ∆N(T_AB_,T_Arr_) for 9°C, 12°C and 15°C were 0.12, 0.06 and 0.03, respectively.

**Figure 5:**
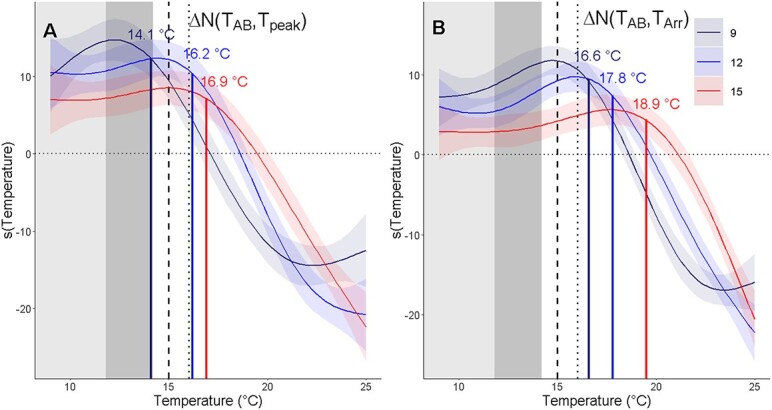
Smoothed fits (s(Temperature)) from GAMs of the relationship between number of individuals at T_AB_ minus those that have reached T_peak_ (∆N(T_AB_,T_peak_)) (A) and relationship between number of individuals at T_AB_ minus those that have reached T_Arr_ (∆N(T_AB_,T_Arr_)) (B) across temperatures for each incubation temperature. Vertical colored lines and associated temperatures indicate the temperature at which ∆N(T_AB_,T_peak_) and ∆N(T_AB_,T_Arr_) begin decreasing with temperature. Plots are fitted smooths and 95% confidence intervals from GAMs. The y-axis units are centered on zero. The estimated degrees of freedom of the smooths for each incubation temperature are: ∆N(T_AB_,T_peak_): 9°C: 4.46, 12°C: 4.49, 15°C: 3.45, ∆N(T_AB_,T_Arr_): 9°C: 5.61, 12°C: 5.18, 15°C: 4.49. Dark gray shaded area indicates temperature range that longfin smelt larvae are cultured at the Fish Conservation and Culture Laboratory (Supplementary Table 3), and light gray shaded areas indicate temperatures where larvae are most abundant in the San Francisco Estuary. Dotted lines indicate the temperature beyond which longfin smelt larvae are no longer observed in the field and dashed lines indicate the temperature where reduced larval growth rates have been measured.

GAM plots showed a negative response for ∆N(T_AB_,T_peak_), indicating that there were more individuals at their upper thermal limit compared relative to those at T_AB_, starting between 16.5–17.7°C, 18.2–19.2°C, and 18.7–20.1°C, for 9°C, 12°C and 15°C incubation temperatures, respectively (Fig. 5A). For ∆N(T_AB_,T_Arr_), GAM plots showed a negative response starting between 18.4°C to 19.1°C, 19.6°C to 20.4°C and 20.5°C to 21.7°C, for 9°C, 12°C and 15°C incubation temperatures, respectively (Fig. 5B). Breakpoints for GAM plots indicated the temperature when ∆N(T_AB_,T_peak_) and ∆N(T_AB_,T_Arr_) began to decrease, that is, when the number of individuals at their upper thermal limit began to increase relative to those at T_AB_. For ∆N(T_AB_,T_peak_), there were breakpoints at 14.1°C, 16.2°C and 16.9°C, for 9°C, 12°C and 15°C, respectively. For ∆N(T_AB_,T_Arr_), there were breakpoints at 16.6°C, 17.8°C and 19.5°C, for 9°C, 12°C and 15°C, respectively.

## Discussion

By transposing a cardiac thermal performance assay typically used for larger fish to newly hatched larvae, and using an activity analysis software, we rapidly assessed cardiac thermal performance of the larval life stage of the imperiled longfin smelt when incubated at different temperatures. We found that initial (*f*_H0_) and peak heart rates (*f*_Hpeak_), and consequently the difference between peak and initial heart rates (∆*f*_Hpeak-H0_) varied across clutches. However, the Arrhenius breakpoint temperature (T_AB_), temperature when heart rate peaks (T_peak_), and temperature when arrhythmia is first observed (T_Arr_) remained relatively similar across clutches. This suggests that while the inherited properties governing cardiac function may have differed, the thermal tolerance range of longfin smelt are similar across clutches. We additionally found that higher incubation temperatures increased T_AB_, T_peak_ and T_Arr_ but that the large and overlapping ranges of these three metrics measured within an incubation temperature rendered the mean and median values of these performance metrics incomplete for informing conservation strategies. We therefore suggest that analysing the proportions of larvae at T_AB_, T_peak_ and T_Arr_ could provide a more appropriate method for identifying T_opt_ and upper thermal limits.

### Use of the cardiac assay

A notable result from the present study was the extremely high individual variation in T_AB_, T_peak_ and T_Arr_ regardless of incubation temperatures; across all incubation temperatures, T_AB_ ranged between 9.4°C and 23.5°C, T_peak_ between 13°C and 25°C and T_Arr_ between 15°C and 25°C. This is consistent with a previous study that found highly variable growth and yolk resorption rates across temperatures in longfin smelt yolk-sac larvae (Yanagitsuru *et al.,* 2021). While some of this variation across individuals can be attributed to parentage, there was low variance attributed to clutch ID for the cardiac performance metrics. This suggests that there is inherently high individual variation in thermal performance within a clutch of SFE longfin smelt. From a life history and evolutionary standpoint, this individual variation in newly hatched larvae is perhaps unsurprising. This life stage has not undergone the expected large mortality event typically observed during the transition from endogenous to exogenous feeding in larvae (Sifa and Mathias, 1987). Therefore, one of the first bottlenecks, where selective pressures can reduce phenotypic variation during recruitment, did not occur (Hjort, 1914). Were this study to be performed at a later life stage, it is possible that this individual variation would be greatly reduced as a result of the inevitable mass-mortality event during the larval life stage. Furthermore, this individual variation among larvae could be adaptive in the dynamic SFE environment where temperature, among other physicochemical properties, can vary greatly on tidal, seasonal and annual timescales (Knowles, 2002), as high phenotypic variation can increase the capacity of a population to persist under a variable environment (Price *et al.,* 2003; Forsman and Wennersten, 2016).

Regardless of the underlying principles resulting in individual variation in longfin smelt larvae thermal tolerance, the extreme variation in cardiac indices complicates the interpretation of the thermal performance metrics. The large range of cardiac indices results in many individuals deviating greatly from the mean values of each of these metrics, and therefore, the mean values would misrepresent a large proportion of the study population. Related to this, individual variation across all three indices resulted in great overlap of the proxy for T_opt_ as measured by T_AB_, and the proxies for upper thermal limits as measured by T_peak_ and T_Arr_, which results in a large mix of individuals that are at appropriate thermal conditions and those that are experiencing thermal stress. For example, in the 9°C incubation temperature group, rearing at mean T_AB_ (14.7°C) would result in 14.0% of individuals at T_AB_, 14.0% having reached T_peak_ and 1.5% having reached T_Arr_.

To interpret cardiac indices for a species and/or life stage with high individual variation, we propose that upper thermal limits are best defined as the temperature where 10% of individuals exhibit T_peak_ or T_Arr_. The 10% thresholds for T_peak_ and T_Arr_ correlate with sublethal and lethal thermal limits for longfin smelt larvae, respectively. For example, in the 9°C incubation temperature group, the 10% threshold for T_peak_ (14.4°C) lies just below the temperature where growth rates are decreased in longfin smelt (15°C) (Yanagitsuru *et al.,* 2021), and the 10% threshold for T_Arr_ (16.9°C) falls within a degree of the temperature where longfin smelt larvae are no longer observed in the field (16°C) (Grimaldo *et al.,* 2017). This makes sense since the 10% threshold for the logistic curves of T_peak_ and T_Arr_ indicate the temperature at which any further warming would lead to a rapid increase in the number of individuals that would reach these indices of upper thermal limit. For management, we suggest under the precautionary principle that the 10% threshold for T_peak_ would ensure that most larvae remain below their upper thermal limit and thus provides a more suitable metric for use in conservation compared to T_Arr_. For example, if the T_Arr_ 10% threshold for the 9°C incubation temperature was applied as a management target, but individuals do indeed begin experiencing thermal stress by T_peak_, then up to 67% of individuals could experience sublethal thermal stress at this temperature. Furthermore, the use of thermal thresholds based on lower incubation temperatures could also ensure most larvae do not experience thermally stressful conditions. For example, if the T_peak_ 10% threshold for the 12°C incubation temperature was applied as a management target, but most wild fish actually incubate at 9°C, then up to 36.5% of wild individuals would hatch into thermally stressful conditions, likely resulting in mortality and recruitment failure.

We also propose that due to the overlapping range of these metrics, T_opt_ can be better approximated as the temperature range where the number or proportion of individuals at T_AB_ is maximized relative to T_peak_ (i.e. maximum ∆N(T_AB_,T_peak_)) for captive-raised individuals. The temperature range where the difference in the proportion or number of individuals at T_AB_ and T_peak_ is maximized across all incubation temperatures correlates with the temperature range where longfin smelt larvae were successfully cultured in captivity (11.9–14.2°C; Supplementary Table 3). In contrast, the temperature range where ∆N(T_AB_,T_peak_) is maximized (12.5–14.0°C depending on incubation temperature) was slightly higher than the T_opt_ inferred from field studies (8–12°C). This discrepancy could be explained by differences in fundamental and realized niches, whereby factors other than temperature such as other physicochemical variables (Dege and Brown, 2003; Grimaldo *et al.,* 2020; Lewis *et al.,* 2020) and food availability (Winder and Jassby, 2011; Lee *et al.,* 2016; Barros *et al.,* 2022; Burris *et al.,* 2022; Fiksen and Reglero, 2022) drive the thermal habitats that organisms occupy in nature (Allen-Ankins and Stoffels, 2017).

### Cardiac thermal performance of larval longfin smelt

Higher incubation temperatures increased mean *f*_H0_, *f*_Hpeak_, ∆*f*_Hpeak-H0_, T_AB_, T_peak_ and T_Arr_. These data suggest that higher incubation temperatures increased the upper bound of cardiac scope, shifted T_opt_ to a higher temperature, and increased upper thermal limits, respectively. Increases to upper thermal limits with higher incubation temperatures is also reflected in the increase of the 10% threshold for T_peak_ and T_Arr_ as well as the breakpoints where our GAMs for ∆N(T_AB_,T_peak_) begin decreasing. Within the temperatures tested, these shifts in cardiac indices suggest that higher incubation temperatures could provide an appreciable tolerance benefit for longfin smelt larvae in a warming world. However, incubation temperature had little influence on the temperature range of maximum ΔN(T_AB,_T_peak_). Taken together, this suggests that the upper thermal limit for a group of longfin smelt larvae is relatively plastic compared to T_opt_. Higher incubation temperatures may increase the resistance of larval longfin smelt to the direct effects of high temperatures but the relatively cooler temperature range of maximum ΔN(T_AB,_T_peak_) would provide the ideal thermal habitat for longfin smelt larvae regardless of their embryonic thermal history. Under climate change, this could mean that the predicted warmer temperatures during embryonic development could provide longfin smelt larvae with higher resistance to the projected increases to temperatures in April and May (Bashevkin *et al.,* 2022), which begin exceeding the 10% T_peak_ thresholds (Brown *et al.,* 2013, 2016). However, continually increasing temperatures during the larval period predicted by climate models (Bashevkin *et al.,* 2022) will result in increasingly larger proportions of larvae being shifted outside of their optimal thermal range. Since maximum ΔN(T_AB,_T_peak_) appears to overestimate field-based optimal thermal habitats, the proportion of longfin smelt larvae falling outside of their ideal temperature range in the SFE are likely to be even higher than would be expected from our results.

The constant incubation temperatures used in this study provide some baseline information on the thermal performance of longfin smelt larvae, but variable thermal regimes more closely reflect what organisms experience in nature. It is well established that variable thermal regimes produce complex phenotypic effects that would not be expected from constant thermal means (Massey and Hutchings, 2021). For example, daily sinusoidal fluctuations in incubation temperature yielded adults with higher critical thermal maxima but smaller size compared to constant mean temperatures in zebrafish (Schaefer and Ryan, 2006). The exact location where longfin smelt spawn is still under investigation (Gross *et al.,* 2022) but areas within the range of potential spawning habitats in the SFE (i.e. San Pablo Bay, Suisun Marsh, Suisun Strait, Carquinez Strait) experience a lightly variable thermal regime during the spawning season, and it is likely that embryos experience daily temperature fluctuations in the SFE. The temperature regime within the spawning season is seasonally variable. Between November and February, temperatures are generally lower with median temperatures typically between 9°C and 15°C, and more stable with temperature fluctuations between ±0.5°C and ±1.5°C daily. In October, March, and April, temperatures increase to median temperatures typically between 12°C and 19°C, with more variable fluctuations typically between ±0.5°C and ±3.5°C (USGS Water Data for USA, https://waterdata.usgs.gov/nwis?). The results of our study most closely represent embryos that are spawned in the cooler and more stable winter months but future studies examining fluctuating incubation temperature regimes would provide a more complete understanding of the thermal habitat requirements of larvae hatched from embryos spawned during the early and late spawning season.

### Management implications

While rising temperature is only one of numerous issues in the SFE, it is important to identify thermal limits for imperiled species living in habitats facing rising temperatures, such as the SFE. We demonstrated that a common cardiac assay has the potential to rapidly identify T_opt_ and upper thermal limits of the larval life stage of a fish species. Our results suggest that it is not possible to provide a single T_opt_ for all, let alone most, individuals from a group of longfin smelt larvae. However, we show that there is a temperature range that maximizes the proportion of individuals at T_opt_ relative to those at their upper thermal limit, which correlates with temperatures at which larvae have been successfully cultured, but may overestimate optimal thermal habitats in the field. We also defined temperature thresholds that would minimize the number of larvae experiencing thermal stress to inform water management policies in the SFE. Our findings suggest that this cardiac assay is a promising tool for quickly identifying T_opt_ for use in culture and for defining thermal thresholds that can be used for field-based management. In particular, this cardiac assay could improve current conservation aquaculture practices and inform supplementation of wild populations.

Conservation aquaculture provides a sustainable supply of research specimens necessary for physiological studies and provides a captive refuge population that can be released for supplementation of wild populations. However, a successful conservation aquaculture program requires the development of culturing methods that can often take years, and successful supplementation efforts requires knowledge of thermal tolerances to identify when and where to release fish (Yanagitsuru *et al.,* 2022). Given the highly limited quantity of research specimens to work with and the time-sensitive nature of protecting imperiled species, it is essential for conservation aquaculture to rapidly identify proper rearing techniques with few individuals to support conservation research. Identifying proper larviculture methods is often the first bottleneck for the successful development of aquaculture for a new species. Traditional grow-out and survival experiments definitively determine the efficacy of culturing protocols, but these studies take time and use large quantities of larvae due to the high densities that larval fish in culture often require. Indeed, for longfin smelt it took over a decade to identify the proper abiotic conditions for culturing larvae (CDWR, 2020). Furthermore, given that population supplementation has already been initiated in endangered delta smelt in the SFE (Baerwald *et al.,* 2023), and considering the population declines and likely impending endangered listing for longfin smelt, it is conceivable that supplementation will also be implemented in longfin smelt. It is therefore critical to quickly develop the culturing tools and obtain the necessary environmental tolerance data for informing future supplementation efforts. While temperature is just one condition, the use of this cardiac assay has the potential to quickly identify the appropriate range of temperatures for captive culture of the larvae of a new species (i.e. maximum ΔN(T_AB,_T_peak_)) with relatively few individuals, and define thermal tolerance thresholds for management efforts, such as supplementation.

Overall, our study demonstrates the utility of this cardiac assay for the early life stages of fishes and our results fill a critical gap in our knowledge of the thermal physiology of longfin smelt. Because mortality during the larval stage influences the success of recruitment in wild fish populations and the success of aquaculture programs, the thermal tolerance metrics that we establish provide a valuable step toward improving longfin smelt conservation in the SFE. The temperatures we defined for captive larviculture through ΔN(T_AB,_T_peak_) and upper thermal limits defined as 10% thresholds can be used to ensure that conservation actions encapsulate appropriate thermal conditions to develop effective rearing protocols for a conservation aquaculture program and to support effective supplementation efforts for this sensitive species. However, it is important to assess thermal tolerance across ontogeny for longfin smelt to inform conservation actions because some species may exhibit ontogenetic variation in thermal tolerance (Komoroske *et al.,* 2014). Furthermore, future studies with this assay across more species and thermal regimes, and comparisons of the relationship between temperatures that maximize ΔN(T_AB,_T_peak_) and 10% thresholds of T_peak_ and T_Arr_ with other thermal performance metrics (e.g. swimming, feeding and survival), are necessary to validate its general utility for fish larvae.

## Supplementary Material

Web_Material_coae004

## Data Availability

Data supporting this paper are available in the Dryad data depository: https://doi.org/10.25338/B8H06D.
