# Supplementary material for: Effects of incubation temperature on the upper thermal tolerance of the imperiled longfin smelt (Spirinchus thaleichthys)
Source: Conserv Physiol. 2024 Feb 10;12(1):coae004. doi: 10.1093/conphys/coae004 (PMC10858410; doi:10.1093/conphys/coae004)
Supplement: Web_Material_coae004 [file web_material_coae004.zip › supplementary data compiled.pdf]

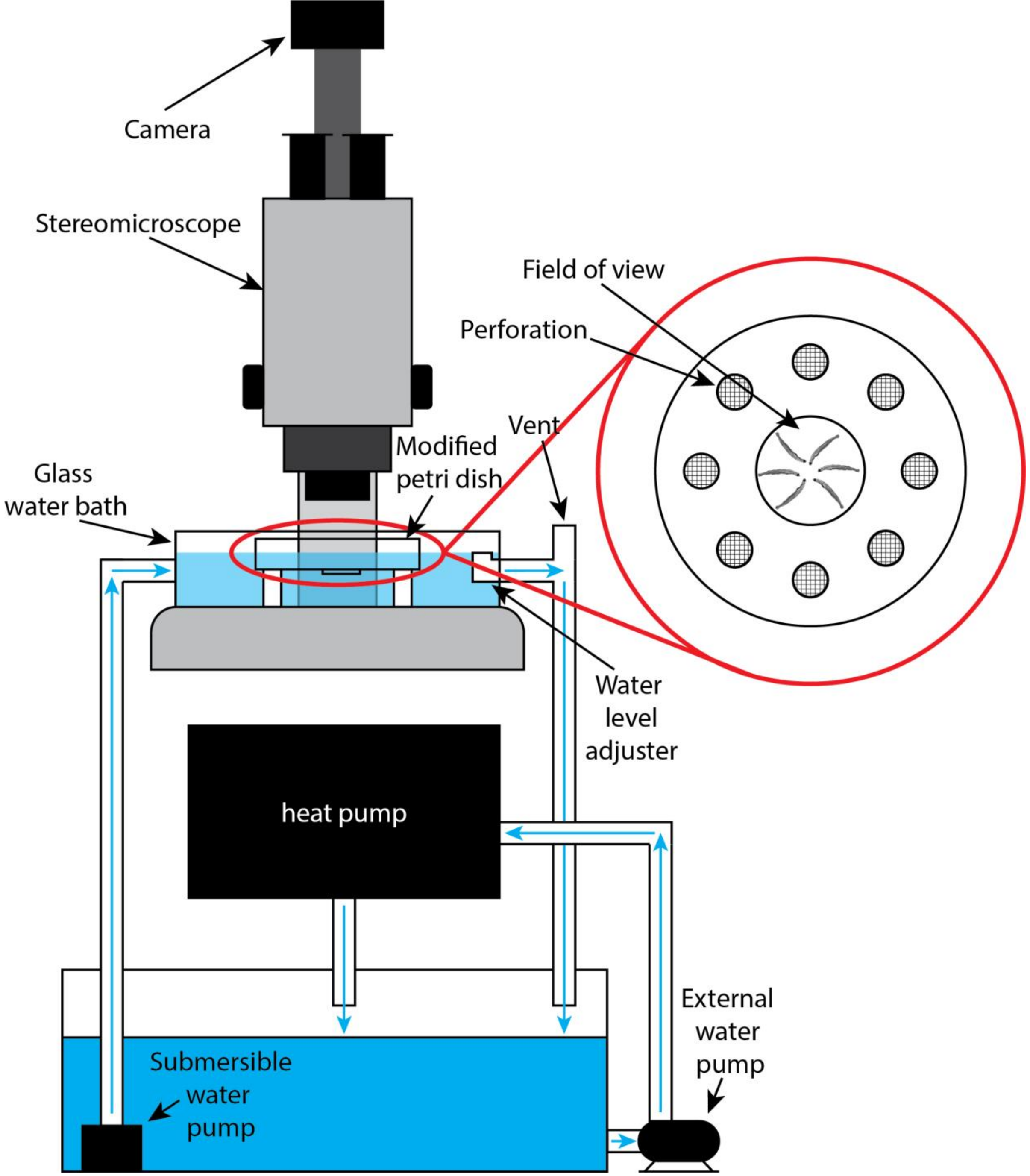

Supplementary Figure 1. Diagram of experimental setup. Experimental setup consisted of a water bath placed under a stereomicroscope fitted with a camera. The water bath had water circulating through it from a temperature-controlled sump. Water temperature within sump was controlled with a heat pump/chiller. Water baths had a PVC elbow within the bath to adjust water level as necessary. The height of petri dishes within the water bath was set with PVC couplers glued to the bottom of the bath. The outflow of the water bath had a vent to equalize pressure to maintain consistent outflow. Petri dishes were modified to have a glass plate in the center to act as a field of view region for placing fish larvae. Each petri dish was perforated and holes covered with 335 µm mesh to allow water from the bath to gently enter the petri dish. Blue arrows indicate the direction of water movement.

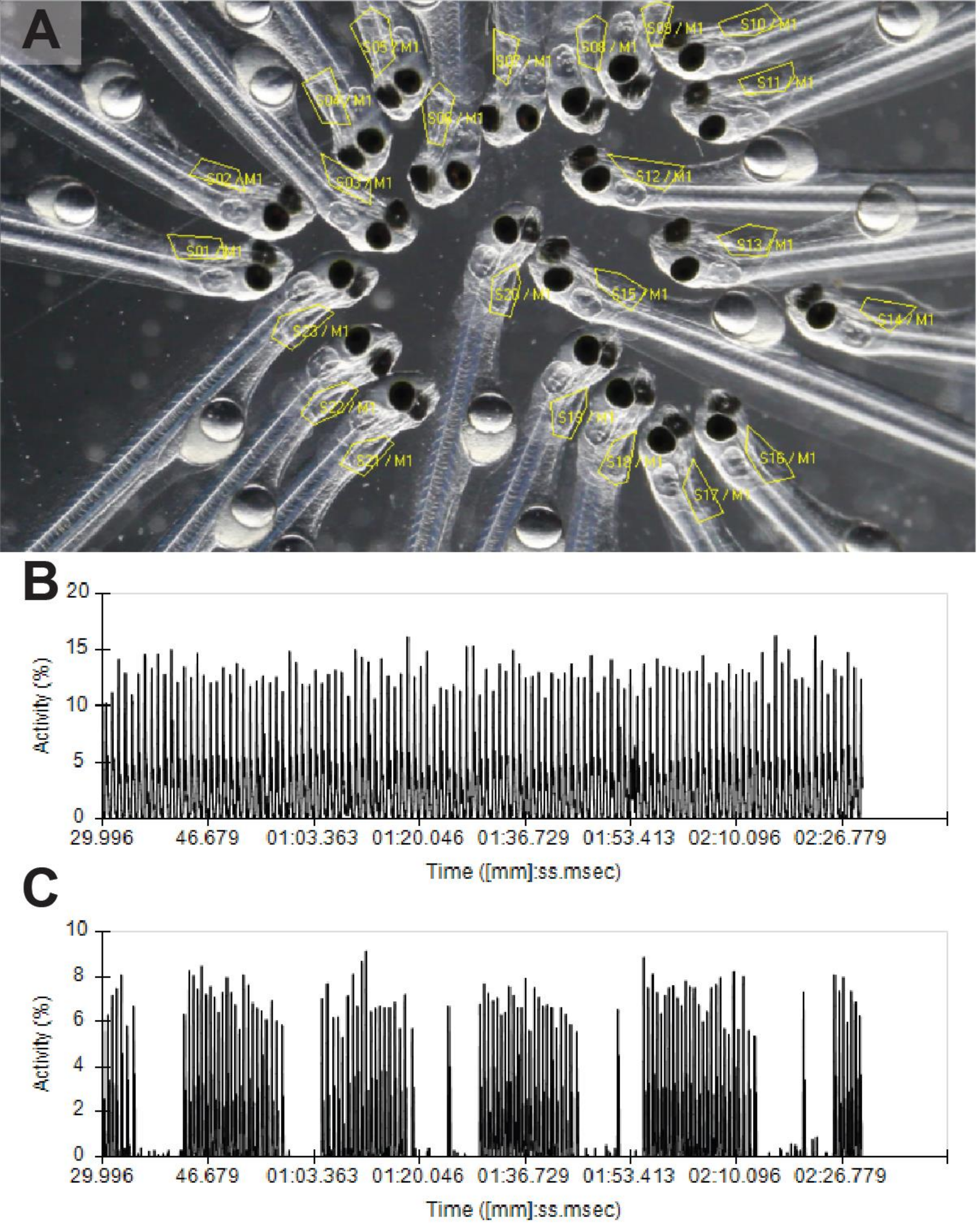

Supplementary Figure 2. Representative raw data from DanioScope (Noldus, Wageningen, Netherlands). Snapshot of a representative video analyzed in DanioScope. Yellow polygons indicate areas where heart rates were measured (A). Representative activity plot of a normal individual's heart rate (B). Representative activity plot of an arrhythmic individual's heart rate (C).

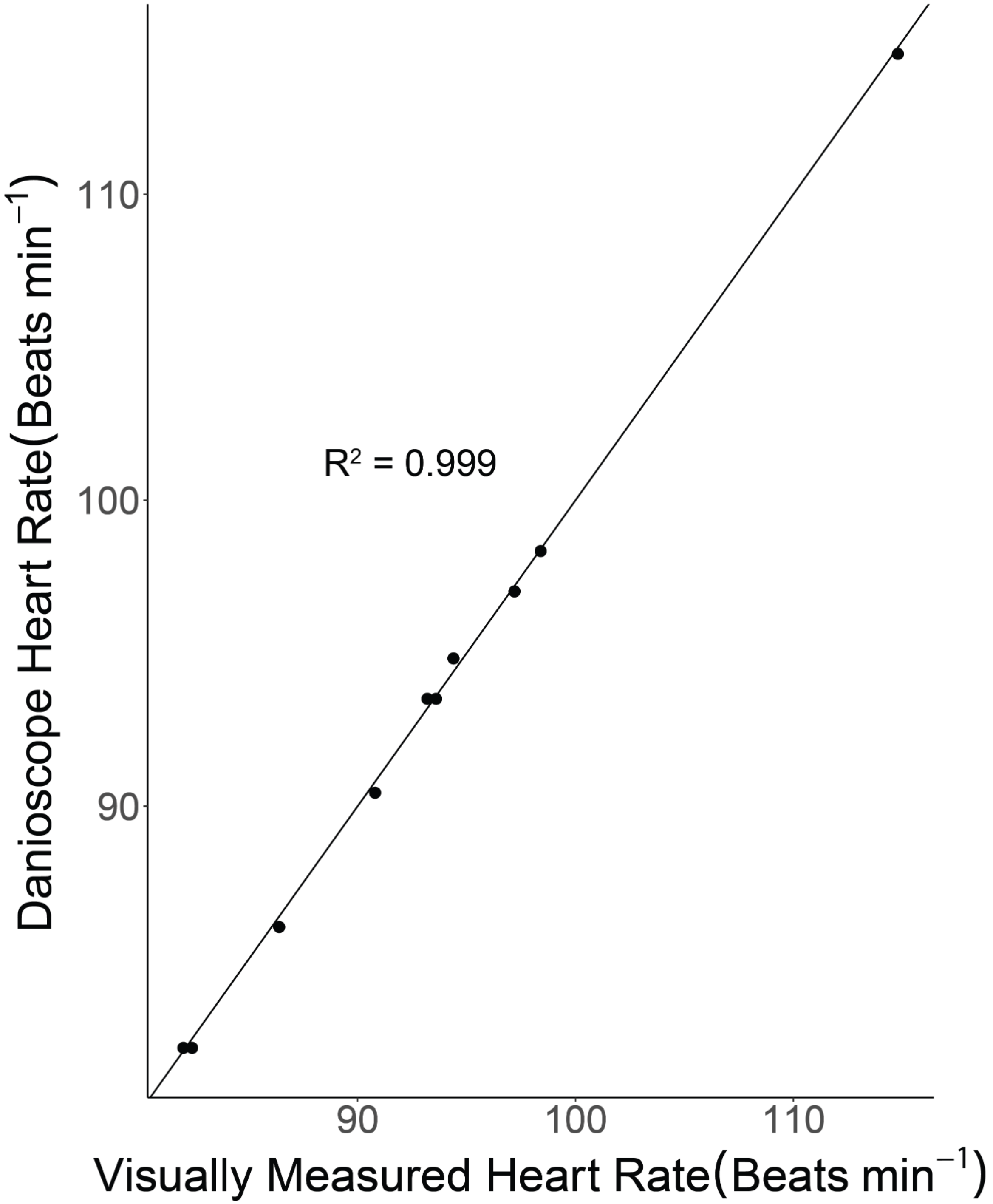

Supplementary Figure 3. Comparison of Danioscope calculated heart rates to visually measured heart rates. Diagonal line indicates 1:1 ratio of Danioscope vs manually calculated heart rates.

Metric	Parameter	Variance	SD	LRT-χ^2 value	P-value
Initial heart rate (f_{H0})	Clutch	8.06	2.84	150.85	<0.001
	Residual	16.77	4.10		
Peak heart rate (f_{Hpeak})	Clutch	174.80	13.22	166.84	<0.001
	Residual	327.50	18.10		
Difference between initial and peak heart rate ($\Delta f_{Hpeak-H0}$)	Clutch	108.20	10.40	124.26	<0.001
	Residual	282.70	16.81		
Rate of increase in heart rate prior to Arrhenius breakpoint temperature (df_H/dT)	Clutch	0.04	0.21	3.81	0.007
	Residual	2.24	1.50		
Arrhenius breakpoint temperature (T_{AB})	Clutch	0.58	0.76	26.29	<0.001
	Residual	6.08	2.47		
Temperature where heart rate reaches a maximum (T_{peak})	Clutch	0.22	0.47	15.97	<0.001
	Residual	3.92	1.98		
Temperature where arrhythmia first occurs (T_{Arr})	Clutch	0.31	0.56	28.66	<0.001
	Residual	2.45	1.56		

Supplementary Table 1. Parameter estimates for random effects of linear mixed models for cardiac function metrics and likelihood ratio test (LRT) values between models including and excluding the random effect of Clutch ID. Bolded p-values indicate statistical significance of random effect (p<0.05).
Model 1: response~incubation + (1|Clutch)
Model 2: response~incubation.

Metric	Parameter	Variance	SD	LRT-χ^2 value	P-value
Temperature where heart rate reaches a maximum (T_{peak})	Clutch ID	0.21	0.46	61.81	<0.001
	Residuals	0.07	0.26		
Temperature where arrhythmia first occurs (T_{Arr})	Clutch ID	0.21	0.46	97.13	<0.001
	Residuals	0.09	0.30		

Supplementary Table 2. Parameter estimates for random effects of generalized linear mixed models for cardiac function metrics and likelihood ratio test (LRT) values between models including and excluding the random effect of Clutch ID. Bolded p-values indicate statistical significance of random effect ($p<0.05$).

Model 1: response~incubation*temperature + (1|Clutch), family = binomial

Model 2: response~incubation*temperature, family = binomial

Year	Temperature Range (°C)
**2020**	11.9-14.1
**2021**	12.4-14.2
**2022**	11.8-13.5
**2023**	12.1-13.6

Supplementary Table 3. Minimum and maximum temperatures in longfin smelt larval tanks at the Fish Conservation and Culture Laboratory between 2020-2023.
